# Dyscalculia, Dyslexia, and Medical Students’ Needs for Learning and Using Statistics

**DOI:** 10.3885/meo.2009.F0000213

**Published:** 2009-02-07

**Authors:** Margaret MacDougall

**Affiliations:** Public Health Sciences Section, Division of Community Health Sciences, University of Edinburgh Medical School

**Keywords:** dyscalculia, dyslexia, inclusive learning resources, recognized learning attributes, statistical literacy, undergraduate medical curriculum

## Abstract

Much has been written on the learning needs of dyslexic and dyscalculic students in primary and early secondary education. However, it is not clear that the necessary disability support staff and specialist literature are available to ensure that these needs are being adequately met within the context of learning statistics and general quantitative skills in the self-directed learning environments encountered in *higher education.* This commentary draws attention to dyslexia and dyscalculia as two potentially unrecognized conditions among undergraduate medical students and in turn, highlights key developments from recent literature in the diagnosis of these conditions. With a view to assisting medical educators meet the needs of dyscalculic learners and the more varied needs of dyslexic learners, a comprehensive list of suggestions is provided as to how learning resources can be designed from the outset to be more inclusive. A hitherto neglected area for future research is also identified through a call for a thorough investigation of the meaning of *statistical literacy* within the context of the undergraduate medical curriculum.

While the majority of higher education institutions provide specialist support services for students with Specific Learning Difficulties (SpLDs), funding limitations have forced the distribution of resources towards addressing language-based difficulties, as these are more representative of learning in a generic sense. In most UK universities, as is the case at the University of Edinburgh, there is no disability support officer in place with a specialist background or professional training in addressing the learning difficulties encountered with mathematics and statistics in particular.^1^

Wherever a similar deficit in specialist support occurs, the medical educator should have a deep awareness of the various obstacles which students with both recognized and unrecognized SpLDs may face as they encounter statistics or other subjects requiring quantitative skills. The need for sensitivity in this respect is particularly acute in an environment with the additional constraints of time and absence of prerequisite skills, as is typically the case when undergraduate medical students engage in short-term research projects involving a substantial level of data preparation, analysis and interpretation.

## Progress in classifying SpLDs

Problems in the classification of SpLDs have been highlighted in the past^2^ but more recently, Nigel Beacham and Clare Trott (both of Loughborough University) have done much to address these problems and raise awareness of dyscalculia and dyslexia in higher education. They have also been involved in the development of DyscalculiUM, a first-line screening test for dyscalculia, available in both paper and electronic forms. This test assumes a fixed threshold score for performance as a means of identifying dyscalculic students.^3^ The need for the screening tool arises from the current lack of effective screening procedures for students who may have dyscalculia only and require subsequent referral to an educational psychologist for a confirmatory diagnosis.^4^

It is encouraging to note that a recent “Phase 3” study assessing the accuracy of this instrument as a diagnostic test for dyscalculia^5^ reports sensitivity and specificity values of 100% when discriminating between dyscalculic and non-dyscalculic students and of 100% and 70% when discriminating between dyscalculic and dyslexic students in particular. Nevertheless, these early results ought to be interpreted tentatively, given that they are based on a total sample of only 30 students, 10 in each group, and the need for reporting corresponding confidence intervals.

The above evidence for continuing progress in diagnosis should be of interest to educators in medical schools when considering the incidence of SpLDs for domiciled and international applicants. For example, based on data provided by the University and College Admissions Service for the UK in 2004^6^, within the cohort for which Medicine and Dentistry was the preferred subject area, 42.3% of those declaring a disability belonged to the category ‘SpLD’. Although the label ‘SpLD’ may be used to refer to a wide variety of learning disabilities, including attention deficit (hyperactivity) disorder, autistic spectrum disorder, dyscalculia, dysgraphia, dyslexia and dyspraxia (developmental coordination disorder), dyslexia is reputed to be the most prevalent learning disability in higher education.^7^,^8^ However, in designing a learning strategy to provide equal opportunities for all undergraduate medical students who are using statistical resources in their research, the educator should not assume that it is exclusively the learning needs of dyslexic students which should be taken into consideration.

Of the SpLDs other than dyslexia, dyscalculia needs particular recognition. Typically, this disability is less well known and educators have an unclear notion (if any) of the distinctive features separating it from dyslexia. Many of the recognized learning attributes symptomatic of these two disabilities are summarized in Table 1.

**Table 1:** Comparison of learning attributes for dyslexia and dyscalculia

SpLD	Recognized learning attributes
Dyslexia^a^	Difficulty with retention and concentration; inefficiency in short-term memory, particularly when reading or listening at considerable speed
	Difficulties in expressing or retrieving what is known or committed to memory
	Phonological processing difficulties (influencing spelling and ability to distinguish words which look similar or causing words and diagrams to be reversed or letters to be inverted or reversed)
	Problems with manipulation of arithmetic and algebraic formulae and of procedures to perform calculations and derive results
	Time-management and organizational problems
	Difficulties with sequencing, including keeping on track with multi-step problems
	Mental and hence physical co-ordination problems (including those relating to listening and note-taking simultaneously and holding several pieces of information in the mind simultaneously in order to arrive at a final solution)
	Tendency in written work to combine words in disjointed form within sentences or omit necessary words from sentences
	Difficulties in identifying the relevant parts of a narrative for translation into the underlying mathematical problem involving formulae; generalizing from concrete to abstract
	Experiencing glare in reading text, particularly with black text on a white background
	Text appearing to move on the page, leading to extreme visual distortion through “see-saw” or “swirl” effects^b^
	The need to see the bigger picture in context prior to understanding the underlying concepts in depth
	The need for space to learn alone and work through or create a personal cognitive representation of a given argument or process
	A heightened potential for creativity
	A preference and marked potential for spatial visualization in grasping concepts within mathematics, statistics and the physical sciences
Dyscalculia	Difficulties in grasping “the most basic skills of numeracy, arithmetic and mathematics” (7), commonly with a distinctive impact on everyday social tasks, such as telling the time

a. See (5), (7), (8), (9), (10), (11) and (12).

b. See (13) for excellent simulations of these effects.

In interpreting Table 1 (especially in the case of dyslexia), it is useful to remember that it is possible for students to have one or more of the symptoms listed without having the corresponding condition. It is the intensity and persistence of these symptoms and, in the case of dyslexia, the number of them that are evident which determines whether a given SpLD is likely and whether it may be appropriate for the student to be referred to an educational psychologist for further assessment. Students may have both dyslexia and dyscalculia, dyslexia only or dyscalculia only, although the latter group is the least common of the three. For students in which both disabilities are present, one of the two is usually identified as the more dominant.^8^

However, it should be noted that some of the learning attributes listed in Table 1 as characteristic of dyslexia may in some employment contexts be more suitably regarded as assets to be honed than difficulties to be surmounted.^9^ For example, the need to see the complex picture may help in providing an accurate diagnosis based on a more holistic understanding of presenting symptoms and risk factors. However, in order to equip medical students with the expertise to apply statistical reasoning responsibly within such a context, considerable groundwork remains to be done in offering an inclusive learning environment.

Moreover, the responsibility of the educator to optimize inclusivity is particularly acute when one considers the tendency of dyslexic learners to disaffection and ultimately, non-compliance when educational systems fail to meet their learning needs.^9^ One can safely assume that similar remarks apply to dyscalculic learners. That the current literature does not make this explicit is likely to be a reflection of problems in differentiating between SpLDs.

McClenaghan's characterization of dyscalculia (Table 1) suggests that it is not exclusively arithmetic or mathematics but numeracy also which may be regarded as a complex hierarchical concept indicative of varying levels of understanding. Furthermore, for the case of numeracy, dyscalculic students are known to encounter difficulties at the lowest of these levels. So, what are “the most basic skills of numeracy” that are particularly problematic for the dyscalculic learner? A credible answer may be found in Sharma and Brazil's definition of dyscalculia as “an inability to conceptualize numbers, number relationships (arithmetic facts) and the outcomes of numerical operations (estimating the answers to numerical problems before actually calculating).”^14^

It is helpful, therefore, to place the most basic skills of numeracy and of arithmetic under the same umbrella and to ask whether there are specific learning skills peculiar to the learning of *statistics* which may place dyscalculic, dyslexic and other categories of medical students at a disadvantage when they are engaged in clinical research projects.

## A hiatus in the literature

The research literature abounds on the topic of supporting dyslexic learners in learning mathematics at primary and early secondary school levels^1^ and there is a growing body of literature related to meeting the corresponding needs of dyscalculic learners.^15^ However, there is a dearth of available literature addressing the specific difficulties experienced by dyscalculic or dyslexic students in learning statistics in higher education, especially within the context of non-numerate disciplines.^1^ Current advice on supporting dyslexic students tends to focus on more generic-type problems relating to sequencing of information, short-term memory difficulties and concentration problems. Also, it appears to be assumed that since the problems with numeracy experienced by dyscalculic learners arise at a basic level, it is unnecessary to conduct research on meeting the needs of such learners at the tertiary level of education.

This deficit in the pedagogical literature is accompanied by a failure to appreciate the uniqueness of applied statistics as a science and therefore to consider the difficulties of dyscalculic and dyslexic students *in context*. Over the last century, statistics has evolved from its position as a sub-discipline of mathematics to become a discipline in its own right. Indeed, Peter Armitage, a distinguished biostatistician and teacher of medical statistics who has served as one of the pioneers in this development remarks,

*“I think it's unreasonable to expect most of the people who need to know about statistics to be able to do very much maths… I can see that an awful lot of people who would be able to understand the purposes of statistical methods, and would be able to use them sensibly, are wasting their time if they really try to go deeply into the maths. In my teaching, I tried to get across purposes, methods, philosophies, and reduce the maths to a minimum.”*^16^

If Armitage's philosophy is to be taken to heart, then current literature on how to make algebraic derivations and proofs more transparent to the dyslexic learner may be of limited use in the teaching of statistics to medical students. Rather, for the medical student, the statistical equivalent of seeing the algebraic problem within the descriptive account may be that of translating a project plan into testable hypotheses. Similarly, in the case of the dyscalculic learner, for example, problems with assessing magnitude and scale may impact on the recognition of statistical significance in terms of p-values and on the interpretation of confidence intervals as an indicator of the accuracy of sample size estimates of treatment effects.

Such possibilities need to be investigated closely if a genuinely inclusive approach is to be taken to developing the lifelong learning skills in critical appraisal which are fundamental to good medical practice. More generally, the challenge for the educator is that of identifying those conceptual areas within medical statistics itself which may prove particularly problematic and which may therefore need to be redressed in the design of more inclusive learning resources.

Using Rasch modeling applied to itemized questionnaires, Watson and Callingham^17^ have validated *statistical literacy* as a complex hierarchical construct defined in terms of thresholds of competence across a continuum of statistical understanding and in terms of a variety of statistical skills defined by specific tasks. However, this construct is specific to secondary education in Australia. Statistical literacy and the corresponding needs of dyscalculic and dyslexic learners within the undergraduate medical curriculum are yet to be understood from an international perspective. Thus, the above construct should be revised to address statistical competencies which undergraduate medical students ought to aspire to at different stages of their curriculum, while allowing for the possibility of variation across countries.

Such work should be informed by an awareness that some dyslexic students may demonstrate mathematical aptitude and have learning difficulties which are predominantly language based.^8^ Although these students may not have encountered any tangible obstacles in learning mathematics within their school curriculum, they may still experience considerable difficulties in engaging with statistics within the context of a research project. For example, identifying pertinent statistical findings which are reported in specialist clinical journals may prove particularly troublesome due to complexities in the style of writing used by the authors and an excess of unfamiliar terms. Such students are also likely to experience difficulties in translating statistical findings into communicable conclusions.

## The design of *inclusive* learning resources

Within the published literature on teaching statistics, there is a lack of evidence to show that the subject-specific learning needs of medical students with dyslexia and dyscalculia have been recognized. This tendency reflects a more general problem in higher education teaching and therefore provides an avenue for future research within a variety of disciplines. However, based on what is already known about recognized learning attributes of students with the above SpLDs (Table 1), considerable headway can be made in the design of appropriate learning resources which take these attributes into account.

In designing learning resources aimed at reducing obstacles to learning in students with SpLDs, the most desirable approach is to aim for inclusivity rather than adapting to accommodate particular needs. Inclusivity, which represents “the best usability for all”^7^ ensures that all users are exposed to the same learning opportunities throughout their learning experience. Further, students without recognized learning difficulties (including the anxious) can benefit from design features that are intended to make the learning and assessment processes less formidable.

Unfortunately, official legislation requiring adaptation to the needs of students with identified SpLDs may fail to guarantee optimal learning opportunities for students with hidden or undeclared disabilities. Thus, for example, the Special Educational Needs and Disability Act of 2001 as defined by the UK parliament for students in pre- and post-16 education simply requires that institutions should make “reasonable adjustments” for disabled students.^18^ However, the educator who is serving the needs of the learner will attach much weight to the following concept of disability captured by Finkelstein:

*“Disability … isn't something that you have. It is something that happens when one group of people create barriers by designing the world only for their style of living.”*^19^

For medical students, the competition for admission to degree courses is so intense that they may be especially hesitant about declaring learning disabilities formally in their applications or even informally when engaging with educators. Learning and assessment tools should be designed from the outset with this possibility firmly in mind. Moreover, the development of fewer but more inclusive learning or assessment tools is likely in the long-term to be a more efficient approach than that of adding on multiple alternative tools retrospectively to meet individual needs as they arise. This observation is especially relevant in a context where a single statistician is employed to meet the needs of the masses with no opportunity in sight for future delegation.

Some of the features which the current author has included in the design of online learning resources to support undergraduate medical students with dyslexia and dyscalculia include:

- Adapting an electronic appointment request system for statistical support to include a sample form for all medical students to read, on which the student making the request declares that they have dyscalculia and dyslexia
- Using ‘previous’, ‘next’ and ‘see that again’ navigational buttons to allow retrieval of previous pages and facilitate retention of an argument in sequence
- Providing a readily accessible online glossary for ease of reference
- Frequently using interactive terms within text via ALT tags which allow students to link to a definition or formula if they are experiencing difficulties with short-term recall
- Providing ‘linking with previous knowledge’ boxes with an identifying icon, to allow students to activate prior learning pages that have content relevant to their current learning
- Using common shading:
  - to highlight key features peculiar to a specific case of a general concept (such as highlighting the distinctive features of the definition of a 90% confidence interval compared to a 95% confidence interval)
  - to address sequencing difficulties when following the thread of an argument
  - to highlight the most salient components of output from a statistical package
- Using colored text boxes, including *‘*rule of thumb’ boxes with an identifying icon, to separate key results from secondary text
- Using a Sans Serif font (such as Arial or Comic Sans) and avoiding upper case letters alone for online text to aid the recognition of words according to their shape
- Developing advanced online searchable indexing systems to facilitate the provision of a window of URLs specifically relevant to the concept the student wishes to explore
- Addressing note-taking problems to enable the dyslexic student to see the bigger picture prior to looking at individual concepts in depth, and to protect the fearful learner:
  - integrating online ‘story books’ with eLearning materials accessed by all students, where these story books take the form of extensive notes on specific concepts (such as the *number needed to treat*) and may be accessed either immediately or if the student prefers, only once a general picture has been acquired of risk notions in context
  - using ‘Want to check the technical details?’ icons with html links to technical explanations of formulae, where exposure to technical complexity is by choice and intellectual curiosity
- Considerably reducing the amount of text which actually appears on any one page on-screen (relative to what could be read in principle)
- Frequently using bullet points to break up paragraphs of text
- Using interactive learning tools involving graphs, identified by ‘Investigate for yourself’ icons, to help the learner visualize statistical concepts hidden behind specific formulae, such as those for confidence intervals, odds ratios and relative risks
- Repeating graphs and tables to remove the need to return to previous pages to verify or visualize observations described in subsequent text
- Using flowcharts to steer students through the required sequence of steps in choosing the right hypothesis tests for their data
- Avoiding flashing objects when using Flash animations
- Presenting the final image as a stationary object on a separate page wherever Flash animations have been designed to help non-dyslexic students view the progressive construction of an image.

The flexible learning environment afforded by some of these features makes for a more personalized approach to learning, which is in and of itself consistent with the dyslexic student's need for space to work things out for themselves.^9^ More generally, it is likely that individual attributes of dyslexia and dyscalculia exist in students with hidden disabilities, such as mental illness and myalgic encephalomyelitis (ME),^20^ and to varying degrees in the wider medical student population. Thus, the above list of innovations, aimed at preventing the learning medium from being a barrier to dyslexic and dyscalculic learners, should also meet the needs of a much wider population.

The reader should additionally be aware of the existence of voice-to-text software, such as *NaturallySpeaking*, text-to-speech software, such as *Jaws* or *Window-Eyes*, symbols-to-words software, such as *Math-Speak,* and mind mapping software, such as *Inspiration.*^21^ In the latter case, the reader may also wish to consult available literature^22^,^23^ for justifications and illustrations relating to the use of mind-maps to eliminate learning barriers.

When considering use of the above software in conjunction with the development of eLearning materials, consultation with a qualified learning technologist prior to the design stage is desirable to decide what provision needs to be made in terms of programming skills and presentation of mathematical expressions and formulae for accessibility by screen-reading software.^24^ Careful planning in the use of programming and software editing facilities can ensure that shaded backgrounds are integral to the design of eLearning materials, thus eliminating the need for the retrospective use of screen overlays to eliminate glare.

The symptoms peculiar to dyscalculia are clearly a concern in the prescribing of appropriate drug dosages for patients. Dyscalculic students are likely to have difficulties in recognizing differences in magnitude between two numbers according to the positions of the decimal point and therefore in recognizing when drug dosages are in the wrong units. However, eDrug,^25^ an online formative assessment tool originally developed to address similar concerns about numeracy skills of undergraduate medical school students in general, may partially address this problem.

## Conclusions

Medical students must be convinced, not only that statistical analyses are indispensable to the sound evaluation of clinical findings but also that the effort required to learn statistics will not have a detrimental effect on their overall research performance. Suggestions have been provided above on how to use more inclusive learning resources so that potential learning barriers can be removed for dyscalculic and dyslexic students in particular. However, it is evident that much research needs to be done on identifying and addressing the specific obstacles that such students are likely to face in the undergraduate medical curriculum when using applied statistics as non-specialist learners.
